# Core-Shell Structured Pt_x_Mo_y_@TiO_2_ Nanoparticles Synthesized by Reverse Microemulsion for Methanol Electrooxidation of Fuel Cells

**DOI:** 10.3389/fchem.2021.667754

**Published:** 2021-04-30

**Authors:** Tianyu Ai, Shuo Bao, Jinlin Lu

**Affiliations:** School of Materials and Metallurgy, University of Science and Technology Liaoning, Anshan, China

**Keywords:** reverse microemulsion method, PtMo alloy, core-shell structure, electrocatalyst, methanol oxidation reaction

## Abstract

The high price of catalyst and poor durability still restrict the development of fuel cells. In this work, core-shell structured Pt_x_Mo_y_@TiO_2_ nanoparticles with low Pt content are prepared by a reverse microemulsion method. The morphologies, particle size, structure, and composition of Pt_x_Mo_y_@TiO_2_ nanoparticles are examined by several techniques such as X-ray Diffraction, X-ray photoelectron spectroscopy and transmission electron microscopy, etc. The Pt_x_Mo_y_@TiO_2_ electrocatalysts show significantly higher catalytic activity and better durability for methanol oxidation than the commercial Pt/C (ETEK). Compared to Pt/C catalyst, the enhancement of the electrochemical performance of Pt_x_Mo_y_@TiO_2_ electrocatalysts can be attributed to the core-shell structure and the shift of the d-band center of Pt atoms, which can weaken the adsorption strength toward CO molecules, facilitate the removal of the CO groups and improve electrocatalytic activity. The development of Pt_x_Mo_y_@TiO_2_ electrocatalysts is promising to reduce the use of noble metal Pt and has a great potential for application in fuel cells.

## Introduction

Direct methanol fuel cell (DMFC) is becoming more and more popular because of its abundant fuel sources, high energy density (6.09 Kwh Kg^−1^), environmental friendliness, high conversion, and low price (Zhao et al., 2011; Zhu et al., 2014; Lin et al., 2020). Due to the disadvantages of the platinum (Pt) group, such as high price, low yield, easy poisoning by oxidized intermediate products and poor durability, it is essential to design a new anode electrocatalyst with high-performance, low price, and high stability. So far, many efforts have been done to achieve the above goals. For example, various nanostructured architectures have been investigated including nanoframe, nanocrystal, nanowires, core-shell, and nanoclusters (Lang et al., 2016; Lu et al., 2016; Kwon et al., 2018; Oh et al., 2018; Liu et al., 2019). Among these nanostructures, core-shell structure is very special and has been widely used in electrocatalysis. Luo et al. (2008) synthesized core-shell structured Au@Pt and Fe_3_O_4_@Au@Pt nanoparticles by using a multiple steps method. The enhancement of catalytic performances for methanol oxidation reaction (MOR) was ascribed to the synergistic effect of oxide core and the shell surface. A core-shell structured Ag@Pt nanoparticle was prepared by the one step method using non-ionic surfactants (Li and Yamauchi, 2013). The activity enhancement is dependent on the adjustment of dendritic Pt shell with large surface area and the good anti-poisoning effect of the Ag core. The core-shell structured Pd@Pt nanoparticles were synthesized using a one-step microwave heating method (Zhang et al., 2010). When Pd/Pt molar ratio was 1:3, the mass activity of Pd@Pt nanoparticles was six times higher than that of commercial Pt/C for MOR. Wang et al. (2019) synthesized PtFe@PtRuFe nanoparticles by using a multi-step solvent method. Due to the core-shell structure and surface alloying, the mass activity of the PtFe@PtRuFe catalysts was improved 1.68 times than that of state-of-the-art PtRu catalysts toward MOR. The core-shell structured Pd-Ni-Pt nanoparticles were synthesized through a wet chemical route (Sneed et al., 2014). The specific activity of Pd-Ni-Pt catalysts for MOR is four times than that of Pd/Pt catalysts. Although various core-shell structured nanocatalysts have been synthesized for MOR of fuel cells, it is still an enormous challenge to develop Pt-based core-shell structured nanoparticles with lower cost, high chemical stability. The electrocatalytic activity and stability of Pt nanoparticles can be further increased by alloying with non-noble metals such as Ni, Fe, and Cu (Ramírez-Caballero et al., 2010; Wang et al., 2011; Zhao et al., 2015). Therefore, it is a challenge to develop a facile method for preparation of high performance and high stability core-shell catalysts with Pt-based alloy. Among these transition metals, Mo has good poisoning tolerance toward MOR at room temperature. Shubina and Koper (2002) verified that the adsorption capacity of CO on PtMo bimetallic surface was weaker than that on pure Pt surface through Density Functional Theory (DFT). However, due to the large negative redox potential of Mo^2+^/Mo couple and the low miscibility of Pt and Mo, it is a very challenging subject to prepare PtMo alloy nanoparticles with small size by using solution-based methods (Liu et al., 2009). In this work, a reverse microemulsion (RME) method is developed to prepare core-shell structured Pt_x_Mo_y_@TiO_2_ nanoparticles. The morphologies, particle size, structure, composition, and performance of Pt_x_Mo_y_@TiO_2_ nanoparticles are investigated in this work. The experimental results show that Pt_x_Mo_y_@TiO_2_ electrocatalysts possess excellent electrocatalytic activity and durability toward MOR.

## Experiment

### Materials

Molybdenum chloride (MoCl_5_, 99.6%), titanium isopropoxide (TiIPO, 96%), isopropanol (C_3_H_8_, 97%), n-heptane (C_7_H_16_, 98.5%), polyoxyethylene (4) lauryl ether (Brij^®^ L4, Mn~362), ammonium hydroxide solution (NH_3_·H_2_O, 25~28%), chloroplatinic acid (H_2_PtCl_6_·6H_2_O, 99.9%), tetraethyl orthosilicate (TEOS, 28.4%), methyl alcohol (CH_3_OH, 99.5%), acetone (C_3_H_6_O, 99.5%), hydrofluoric acid (HF, 40%), Nafion solution (5% in isopropanol and water) were purchased from AiKe reagent without further treatment.

### Synthesis of Pt_x_Mo_y_@TiO_2_ Nanoparticles

The Pt_x_Mo_y_@TiO_2_ nanoparticles were synthesized by referencing a previously modified method which was reported by Sean T. (Hunt et al., 2016a). In a typical synthesis process, 8 mL C_12_H_28_O_4_Ti and 4 mL MoCl_5_ were added into the mixed solutions with 120 mL C_7_H_16_ and 55 mL Brij^®^ L4, which formed the transition metal precursor alcohol solution. A certain amount of H_2_PtCl_6_ · 6H_2_O aqueous solution with 45 mL C_7_H_16_ and 7.5 mL Brij^®^ L4 were injected into the above solution drop by drop. After stirring the mixture for 4.2 h, 1.0 mL TEOS was added quickly. After 16.5 h, 300 mL methanol was added and stirred for 15 min, then lay up for 1 h at least. The white precipitate was collected by centrifugal, washed with acetone and dried under vacuum at 60°C overnight. Then white powers were heated at a rate of 2°C min^−1^ to 870°C and maintained for 300 min under 120 cm^3^ min^−1^ of H_2_ and 30 cm^3^ min^−1^ of CH_4_, then the SiO_2_@Pt_x_Mo_y_@TiO_2_ nanoparticles were obtained. After slowly cooling to room temperature, the SiO_2_@Pt_x_Mo_y_@TiO_2_ nanoparticles were etched by HF and ethanol for 15 h to remove the SiO_2_ outer shell. Finally, the Pt_x_Mo_y_@TiO_2_ nanoparticles were collected by centrifugation and washed with ethanol and water and dried under vacuum at 60°C overnight.

### Characterization

The phase of nanoparticles was characterized by X-ray Diffraction (XRD) using Cu Kα radiation. The diffraction patterns were stored from 2θ = 10–90° with a scan rate of 5° s^−1^. The surface composition and structure of as-prepared catalysts under ultra-high vacuum were characterized by X-ray photoelectron spectroscopy (XPS) with a monochromatic Al X-ray source (Al-KR, 1486.8 eV). All spectra were corrected by referencing the adventitious C 1s signal to 284.7 eV. The morphology and size of nanoparticles were characterized under instrument operating at 200 kV by Transmission Electron Microscopy (TEM). The samples were dispersed in acetone solution, dropped into copper TEM grids with carbon film, cooled to room temperature. The composition of the nanoparticles was analyzed by an energy-dispersive X-ray (EDX) spectroscope attached to the TEM.

Electrochemical measurements were carried out by using a common three-electrode cell method on the Autolab (Metrohm, PGSTAT 302N) instrument at room temperature. A glassy carbon electrode (GCE) with a diameter of 5 mm was used as the working electrode (WE), a 10 × 20 mm^2^ platinum sheet was used as the counter electrode, and a silver chloride was used as the reference electrode (Ag/AgCl). Catalyst inks consisted of 10 mg Pt_x_Mo_y_@TiO_2_ nanoparticles and 2 mL, 0.5% Nafion/isopropanol, which was treated by ultrasound for 30 min. Finally, 10 μL catalyst inks were dropped on the GCE surface and dried in air at room temperature.

## Results and Discussion

**Figure 1:**
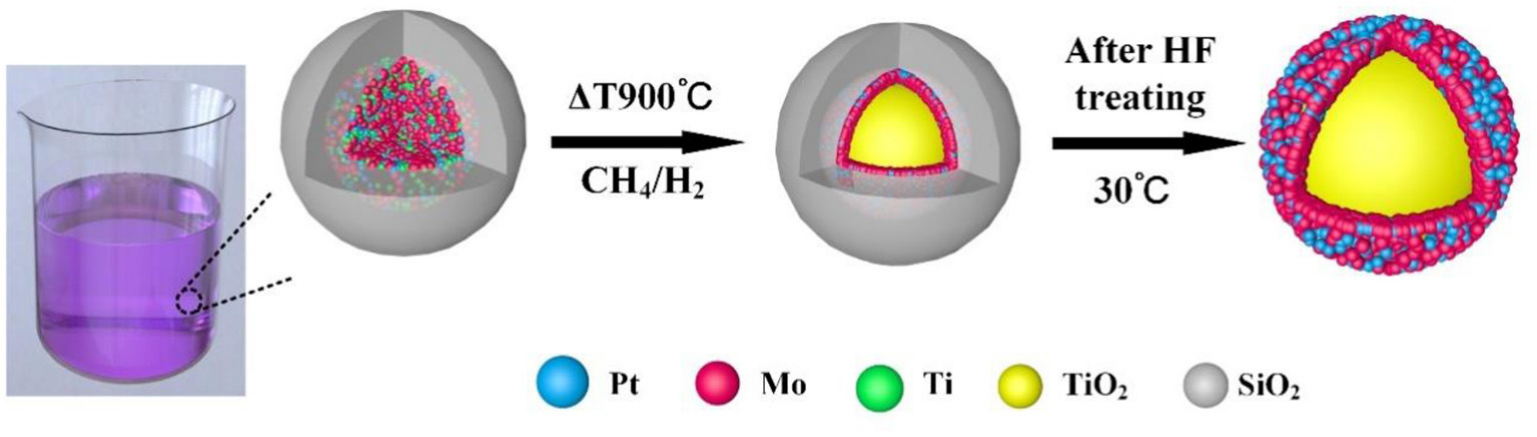
Schematic diagram of the synthesis of Pt_x_Mo_y_@TiO_2_ nanoparticles.

Core-shell structured Pt_x_Mo_y_@TiO_2_ nanoparticles are obtained by using the RME method. The synthesis procedure is described in Figure 1. First, the precursor structure is formed through the RME process. The resulting composite particles are subjected to a high-temperature reduction process, wherein the SiO_2_ coating serves as a hard template to prevent nanoparticles sintering. Finally, core-shell structured Pt_x_Mo_y_@TiO_2_ nanoparticles are obtained by dissolving the SiO_2_ shell using an aqueous HF solution.

**Figure 2:**
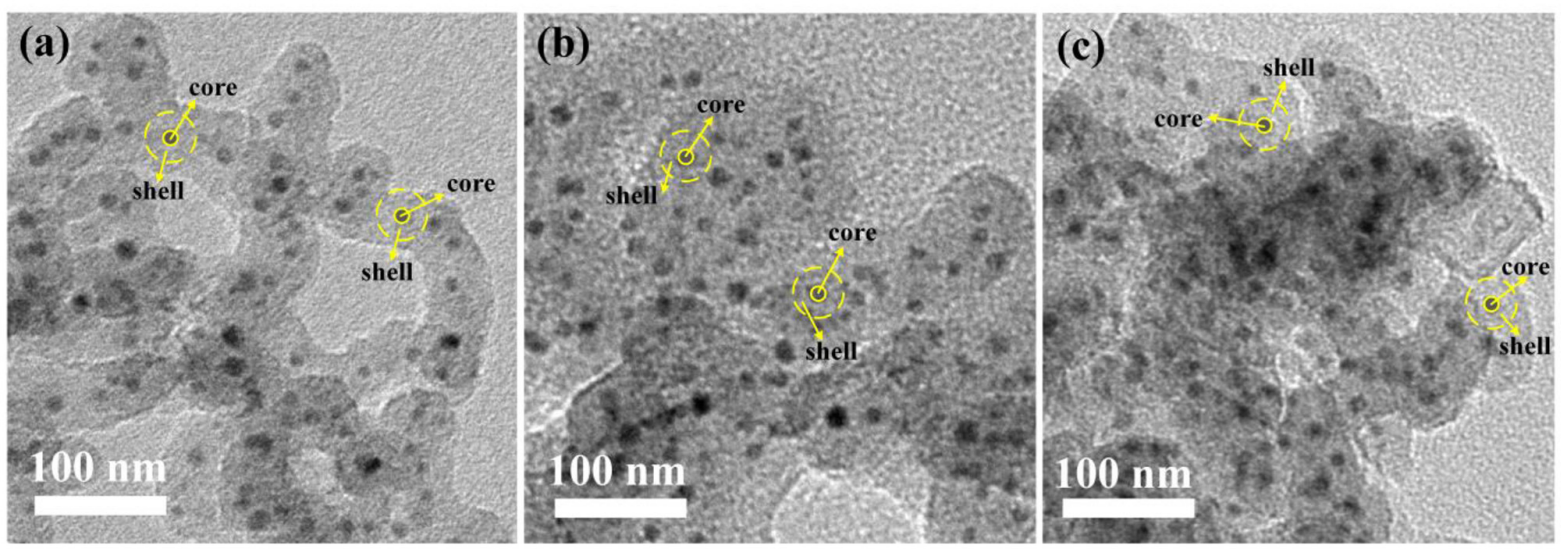
TEM images with high magnification inset of **(a)** SiO2@Pt0.5Mo0.5@TiO2, **(b)** SiO2@Pt0.25Mo0.75@TiO2, and **(c)** SiO2@Pt0.2Mo0.8@TiO2.

After high temperature sintering, TEM images of SiO_2_@Pt_x_Mo_y_@TiO_2_ nanoparticles are shown in Figure 2. The images were mared with yellow dotted line to clarify the different parts for shell and core. The dark dot in the center represents the Pt_x_Mo_y_@TiO_2_ core and the light shadow in the outer represents the SiO_2_ shell. It can be found that all the dark dots are coated with thick shells. And the Pt_x_Mo_y_@TiO_2_ nanospheres in the core show uniform distribution without obvious agglomeration.

**Figure 3:**
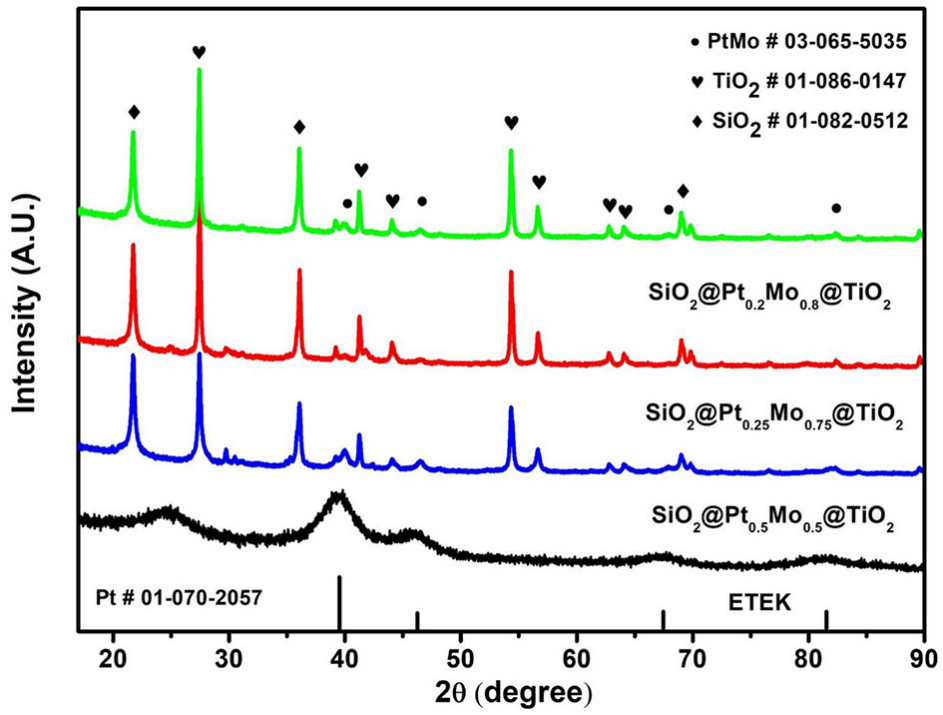
XRD patterns of ETEK, SiO2@Pt0.5Mo0.5@TiO2, SiO2@Pt0.25Mo0.75@TiO2, and SiO2@Pt0.2Mo0.8@TiO2.

The crystal structures of SiO_2_@Pt_x_Mo_y_@TiO_2_ and ETEK nanoparticles are characterized by the XRD pattern. As shown in Figure 3, the diffraction peaks of SiO_2_ (PDF#01-082-0512) are located at 21.8, 35.9, and 69.0°. A set of diffraction peaks for TiO_2_ at 2θ of 27.4, 41.2, 44.0, 54.3, and 56.6° indicate the existence of TiO_2_ (PDF#01-086-0147) in the compounds. The diffraction peaks of PtMo alloy (PDF#03-065-5035) are located at 39.93, 46.43, 67.78, and 81.65°. Each of them corresponds to a crystal face, which is (111), (200), (220), and (311) facets of face-centered cubic (fcc) structure(Chen and Pan, 2009; Zhang et al., 2019). Because the content of PtMo is much lower than TiO_2_ and SiO_2_, the intensities of PtMo diffraction peaks are much lower than TiO_2_ and SiO_2_.

**Figure 4:**
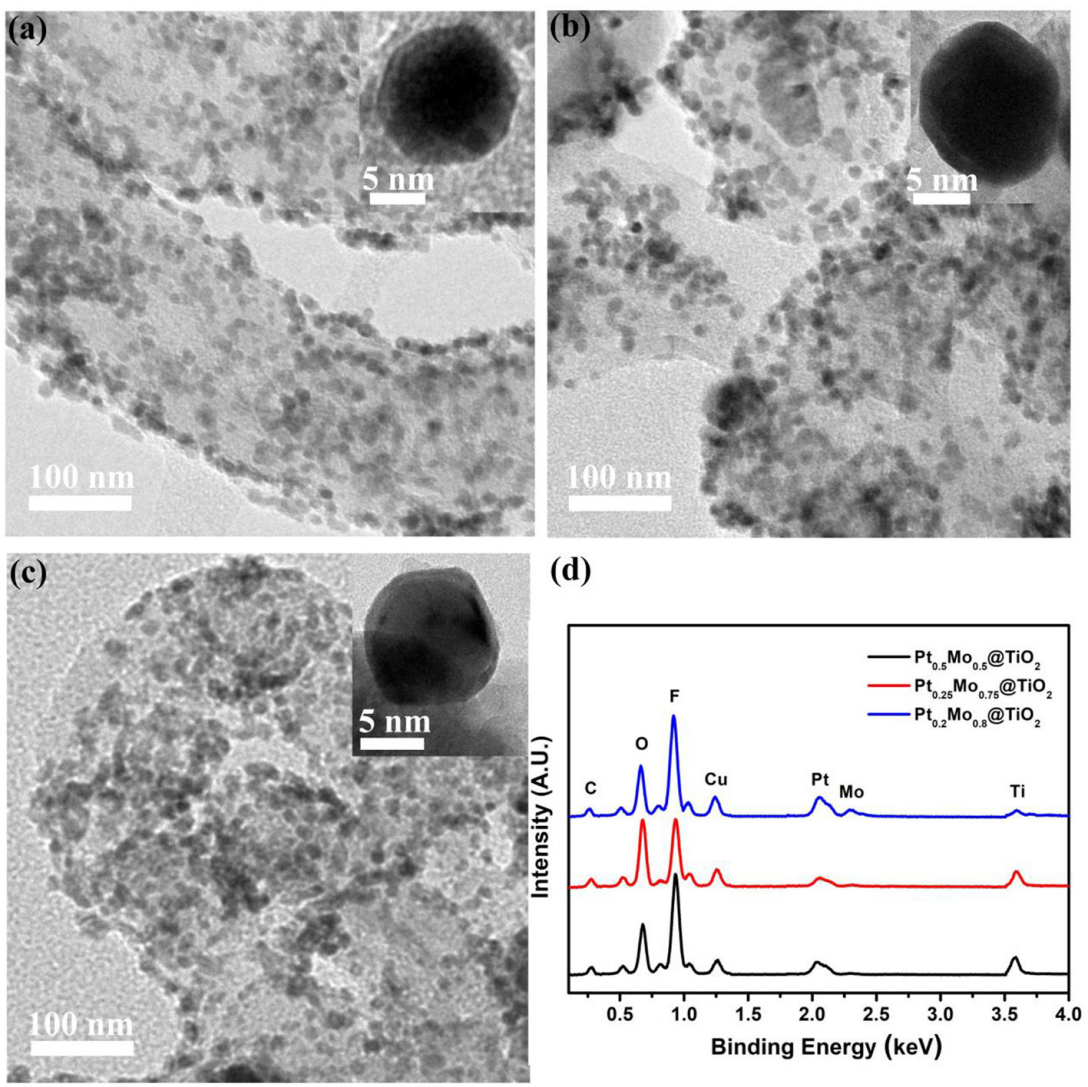
TEM images with high magnification inset of **(a)** Pt0.5Mo0.5@TiO2, **(b)** Pt0.25Mo0.75@TiO2 and **(c)** Pt0.2Mo0.8@TiO2, and **(d)** the corresponding EDX spectrums.

The morphology and composition of the Pt_x_Mo_y_@TiO_2_ nanoparticles without silica shell are further characterized by TEM combined with EDX, as shown in Figure 4. The nanoparticle sizes of Pt0.5Mo0.5@TiO2, Pt0.25Mo0.75@TiO2, and Pt0.2Mo0.8@TiO2 are all about 12~15 nm with uniform distribution. Inset high magnification pictures clearly show that PtMo alloy is uniformly coated with TiO_2_. The EDX spectra results of different nanoparticle components are shown in Figure 4d. The EDX images show that the existence of Pt, Mo and Ti elements, and the intensity of Mo peak increases with increasing the Mo content in the precursor solution. The existence of Cu elements comes from the copper mesh used in the preparation of TEM samples.

**Figure 5:**
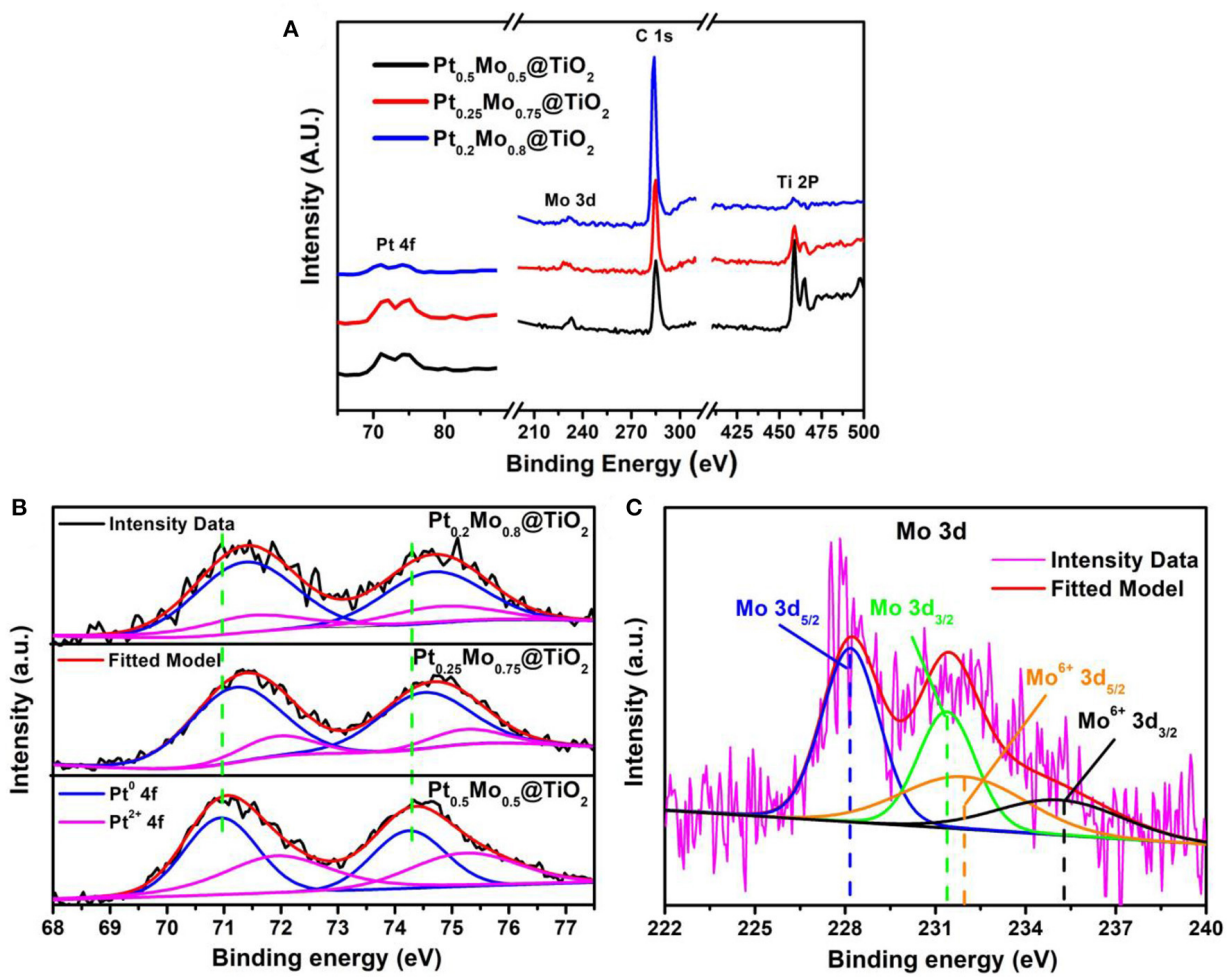
**(A)** Survey scan, **(B)** Pt 4f spectrum of Pt0.5Mo0.5@TiO2, Pt0.25Mo0.75@TiO2, and Pt0.2Mo0.8@TiO2, **(C)** Mo 3d spectrum of Pt0.25Mo0.75@TiO2.

The surface element composition and chemical state of Pt_x_Mo_y_@TiO_2_ nanoparticles are further investigated by XPS measurements. It can be found that three elements of Pt, Mo, and Ti exist in XPS survey, as shown in Figure 5A. The Pt 4f region of the Pt_x_Mo_y_@TiO_2_ samples can be divided into two pairs of doublets, as shown in Figure 5B. The Pt0.5Mo0.5@TiO2 nanoparticles exhibit a pair of Pt 4f7/2 signal centered at 71.0 and 74.3 eV, which is consistent with the metallic Pt (Cao et al., 2013; Hunt et al., 2016b). The weak doublet peaks at 72.1 and 75.4 eV can be assigned to the Pt oxides (Tang et al., 2018). The Pt0.25Mo0.75@TiO2 and Pt0.2Mo0.8@TiO2 also show two doublet peaks that can be associated with metallic Pt and Pt oxides. Obviously, a pair of peaks at 71.2 and 74.5 eV are associated with the metallic Pt of Pt0.25Mo0.75@TiO2, another pair of peaks at 71.4 and 74.7 eV are associated with the metallic Pt of Pt0.2Mo0.8@TiO2. Compared with Pt0.2Mo0.8@TiO2 samples, a large negative shift in Pt 4f7/2 binding energy is observed in Pt0.5Mo0.5@TiO2 and Pt0.25Mo0.75@TiO2 samples. Due to the downshift in Pt 4f binding energy, more electrons will be transferred to Pt in Pt0.5Mo0.5@TiO2 and Pt0.25Mo0.75@TiO2 samples, which can weaken the Pt-CO_ads_ and promote the C-H cleavage on Pt sites (StamenkovIc et al., 2007; Stephens et al., 2011; Dubau et al., 2015). Figure 5C shows the Mo 3d spectra of the Pt0.5Mo0.5@TiO2 sample. The results show that the strong double peaks at 228.2 and 231.4 eV can be assigned to metallic Mo, the weak doublet peaks at 231.7 and 234.9 eV can be assigned to the Mo oxides. This indicates that Mo exists in the form of PtMo alloy in the Pt0.25Mo0.75@TiO2 samples, which is consistent with the XRD results.

**Figure 6:**
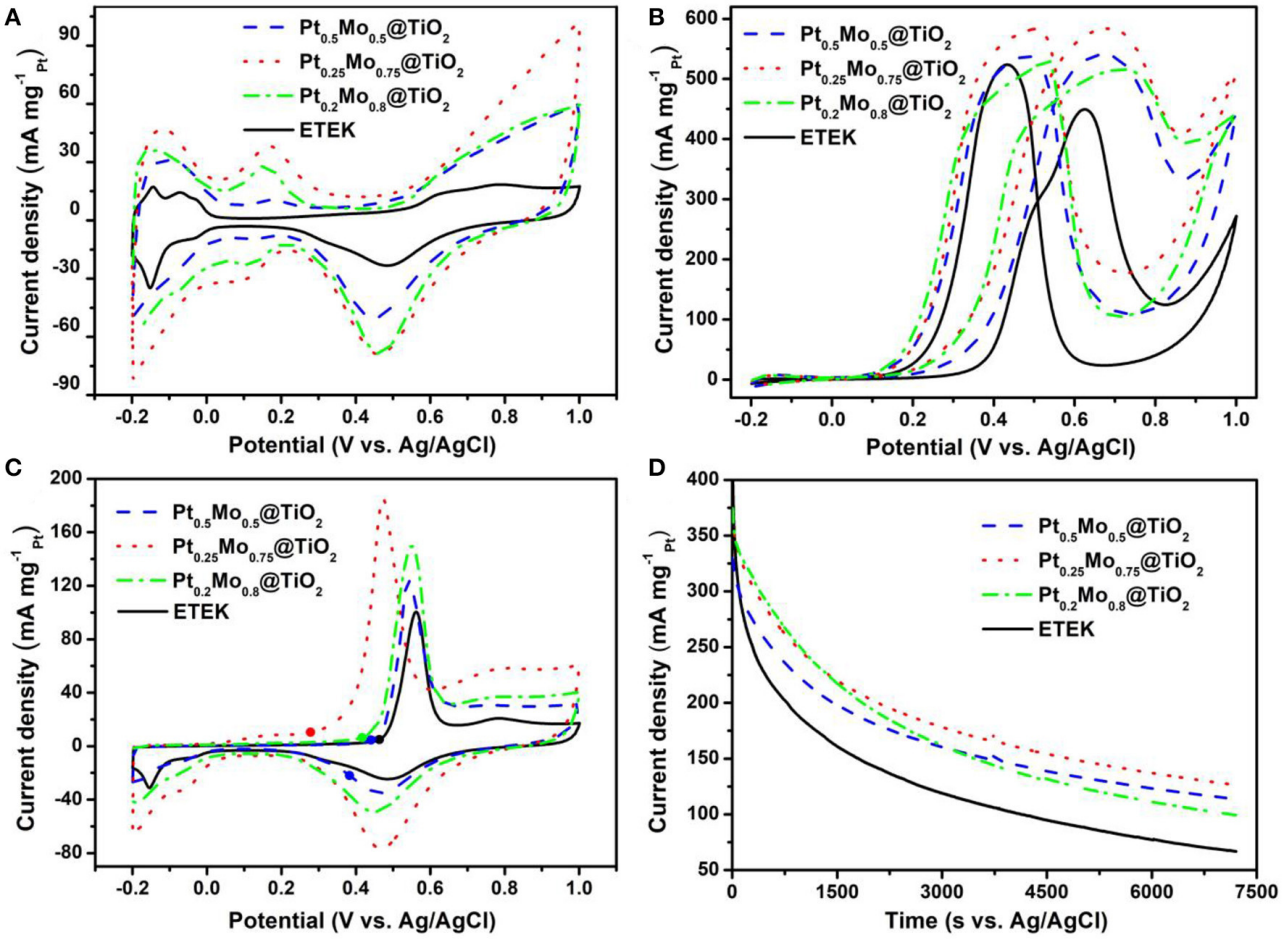
CV of the catalysts **(A)** in N_2_-saturated 0.5 M H_2_SO_4_ solution, **(B)** in N_2_-saturated 0.5 M H_2_SO_4_ + 1.0 M CH_3_OH solution and **(C)** CO stripping voltammograms in 0.5 M H_2_SO_4_ solution at 25°C with a scan rate of 50 mV s^−1^, **(D)** CA curves of the catalysts in N_2_-saturated 0.5 M H_2_SO_4_ + 1.0 M CH_3_OH solution at 25°C.

The electrocatalytic activities of ETEK, Pt0.5Mo0.5@TiO2, Pt0.25Mo0.75@TiO2, and Pt0.2Mo0.8@TiO2 electrocatalysts are examined at room temperature in N_2_-saturated 0.5 M H_2_SO_4_ with or without 1.0 M CH_3_OH solutions, as shown in Figure 6. The electrochemical surface area (ECSA) is an important parameter, which is usually measured by the hydrogen adsorption/desorption area in the cyclic voltammograms (CV) curves and assumed to be 210 μC cm^−2^ for the adsorption of a hydrogen monolayer (Lim et al., 2009). The typical CV of different electrocatalysts in 0.5 M H_2_SO_4_ solution are displayed in Figure 6A. The scan rate is 50 mV s^−1^ in the potential range of −0.2–1.0 V. The comparison of ECSA between ETEK and as-prepared electrocatalysts is shown in Figure 6A and the corresponding electrochemical parameters are shown in Table 1. The Pt0.25Mo0.75@TiO2 electrocatalyst has a higher ECSA (593.9 cm^2^ ${\text{mg}}_{\text{Pt}}^{-1}$), about 3.1 times higher than that of ETEK (193.3 cm^2^ ${\text{mg}}_{\text{Pt}}^{-1}$). The high active surface area of Pt0.25Mo0.75@TiO2 is attributed to the small size and the synergistic effect between Pt and Mo. Higher ECSA tends to provide more active sites for hydrogen adsorption/desorption, which is very favorable for MOR.

**Table 1:** The properties and electrochemical parameters of the different catalysts.

**Catalysts**	**ECSA (cm^**2**^${\text{mg}}_{\text{Pt}}^{-1}$)**	**Onset potential (V)**	**Peak potential (V)**	**Peak current (mA ${\text{mg}}_{\text{Pt}}^{-1}$)**	**Ratio (*I_***f***_/I_***b***_*)**
ETEK	193.3	0.23	0.63	449.37	0.86
Pt0.5Mo0.5@TiO2	275.2	0.11	0.64	540.34	1.01
Pt0.25Mo0.75@TiO2	593.9	0.12	0.68	583.86	1.00
Pt0.2Mo0.8@TiO2	471.6	0.18	0.7	515.74	0.97

A negative onset potential and a higher peak current density of MOR are also needed for a good catalyst (Lu et al., 2012). As shown in Figure 6B, the onset potential of the Pt0.25Mo0.75@TiO2 catalyst for MOR is 0.12 V, which is more negative than that of ETEK (0.23 V). The results indicate that MOR can occur more easily on the Pt0.25Mo0.75@TiO2 than on the ETEK. To gain further insights into the activities of different catalysts, the electrocatalytic activities of ETEK, Pt0.5Mo0.5@TiO2, Pt0.25Mo0.75@TiO2, and Pt0.2Mo0.8@TiO2 electrocatalysts for MOR in N_2_-saturated 0.5 M H_2_SO_4_ with 1.0 M CH_3_OH solution are examined at room temperature as shown in Figure 6B. The test parameters of Figure 6B are the same as Figure 6A. The peak current density of ETEK for the MOR is 449.37 mA ${\text{mg}}_{\text{Pt}}^{-1}$. However, the peak current densities of Pt0.5Mo0.5@TiO2, Pt0.25Mo0.75@TiO2, and Pt0.2Mo0.8@TiO2 samples for the MOR are 1.2 times (540.34 mA ${\text{mg}}_{\text{Pt}}^{-1}$), 1.3 times (583.86 mA ${\text{mg}}_{\text{Pt}}^{-1}$), and 1.5 times (515.47 mA ${\text{mg}}_{\text{Pt}}^{-1}$) than that of ETEK. The improvement of catalyst activity is due to the alloying effect of PtMo, which results in the shift of the d-band center of the surface Pt atoms and the increase of MOR activity (Han et al., 2015).

The I_f_/I_b_ ratio represents the tolerance of CO on the platinum surface, in which If is the forward peak current and I_b_ is the reverse peak current (Hassan et al., 2015). The I_f_/I_b_ values of the Pt0.5Mo0.5@TiO2, Pt0.25Mo0.75@TiO2, Pt0.2Mo0.8@TiO2, and ETEK are calculated to be 1.01, 1.00, 0.97, and 0.86, respectively (Table 1), implying that the Pt_x_Mo_y_@TiO_2_ electrocatalysts have a high tolerance to CO poisoning species. The anti-poisoning of CO is further demonstrated by CO stripping voltammograms in 0.5 M H_2_SO_4_ solution at 25°C with a scan rate of 50 mV s^−1^. As shown in Figure 6C, the onset potentials of Pt0.5Mo0.5@TiO2, Pt0.25Mo0.75@TiO2, and Pt0.2Mo0.8@TiO2 are 0.28, 0.39, and 0.44 V. However, the onset potential of ETEK is 0.46 V, illustrating that the pre-adsorbed CO on Pt0.5Mo0.5@TiO2 and Pt0.25Mo0.75@TiO2 catalysts is easier to be oxidized than that on ETEK. The bi-functional mechanism could further explain the resisted CO poisoning of PtxMoy@TiO2 electrocatalyst in theory. The bi-functional mechanism could be summarized as follows (Levy and Boudart, 1973; Wang et al., 2006; Lu et al., 2016):

(1) $$\begin{array}{l} \text{Pt}+\text{C}{\text{H}}_{3}\text{OH}\to \text{PtC}{\text{O}}_{\text{ads}}+4{\text{H}}^{+}+{4\text{e}}^{-} \end{array}$$

(2) $$\begin{array}{l} \text{Mo}+{\text{H}}_{2}\text{O}\to \text{Mo}{\left(\text{OH}\right)}_{\text{ads}}+{\text{H}}^{+}+{\text{e}}^{-} \end{array}$$

(3) $$\begin{array}{l} \text{PtC}{\text{O}}_{\text{ads}}+\text{M}{\text{o}\left(\text{OH}\right)}_{\text{ads}}\to \text{C}{\text{O}}_{2}+\text{Pt}+\text{Mo}+{\text{H}}^{+}+{\text{e}}^{-} \end{array}$$

Mo can activate the interfacial water and generate OH species at low potential to facilitate the oxidation of intermediates species, such as CO_ad_, which is evidenced by the CO_ad_ stripping voltammograms. The electrocatalytic stabilities of Pt_0.5_Mo_0.5_@TiO_2_, Pt0.25Mo0.75@TiO2, Pt_0.2_Mo_0.8_@TiO_2_, and ETEK were tested under 0.6 V for 7200 s in N_2_-saturated 0.5 M H_2_SO_4_ solution containing 1.0 M CH_3_OH solutions, as shown in Figure 6D. In the early stage, the current density decreases sharply because the electrocatalyst surfaces are poisoned (Wang et al., 2014; Jin et al., 2020). As time goes by, the current decreases slowly. After 7200 s, the current of pre-prepared electrocatalyst is also higher than that of commercial ETEK. The polarization currents of Pt_0.5_Mo_0.5_@TiO_2_, Pt0.25Mo0.75@TiO2, and Pt_0.2_Mo_0.8_@TiO_2_ are 113.8, 126.4, and 99.5 mA ${\text{mg}}_{\text{Pt}}^{-1}$, respectively, but ETEK is only 66.8 mA ${\text{mg}}_{\text{Pt}}^{-1}$.

The comparisons between some papers published recently and this work are tabled in Table 2. It can be seen clearly that the Pt0.25Mo0.75@TiO2 electrocatalyst in this work shows the highest mass activity for MOR in the similar testing conditions.

**Table 2:** Comparisons of the MOR performance for Pt based catalysts in recently published papers.

**References**	**Catalyst**	**ECSA (cm^**2**^${\text{mg}}_{\text{Pt}}^{-1}$)**	**Electroolyte**	**Mass activity (mA ${\text{mg}}_{\text{Pt}}^{-1}$)**	**Methods**
Zhu et al. (2014)	Pt/NC_x_-TiO_2_-2	—	0.5 M H_2_SO_4_ +1.0 M CH_3_OH	382.2	Microwave-assisted method
Tang et al. (2018)	PtNiCu ERDS	615	0.5 M H_2_SO_4_ +1.0 M CH_3_OH	—	One-pot method
Lu et al. (2012)	Pt/WC/C_10_	423.6	0.5 M H_2_SO_4_ +1.0 M CH_3_OH	313	Improved impregnation method
Levy and Boudart (1973)	Pt@WC/OMC	—	0.5 M H_2_SO_4_ +1.0 M CH_3_OH	367.5	Pulse-microwave polyol method
Wang et al. (2006)	Pt/TiO_2_@NC-NCTs-7	826	0.5 M H_2_SO_4_ +1.0 M CH_3_OH	577	Solvent heating method
This work	Pt0.25Mo0.75@TiO2	593.5	0.5 M H_2_SO_4_ +1.0 M CH_3_OH	583.86	Reverse microemulsion method

**Figure 7:**
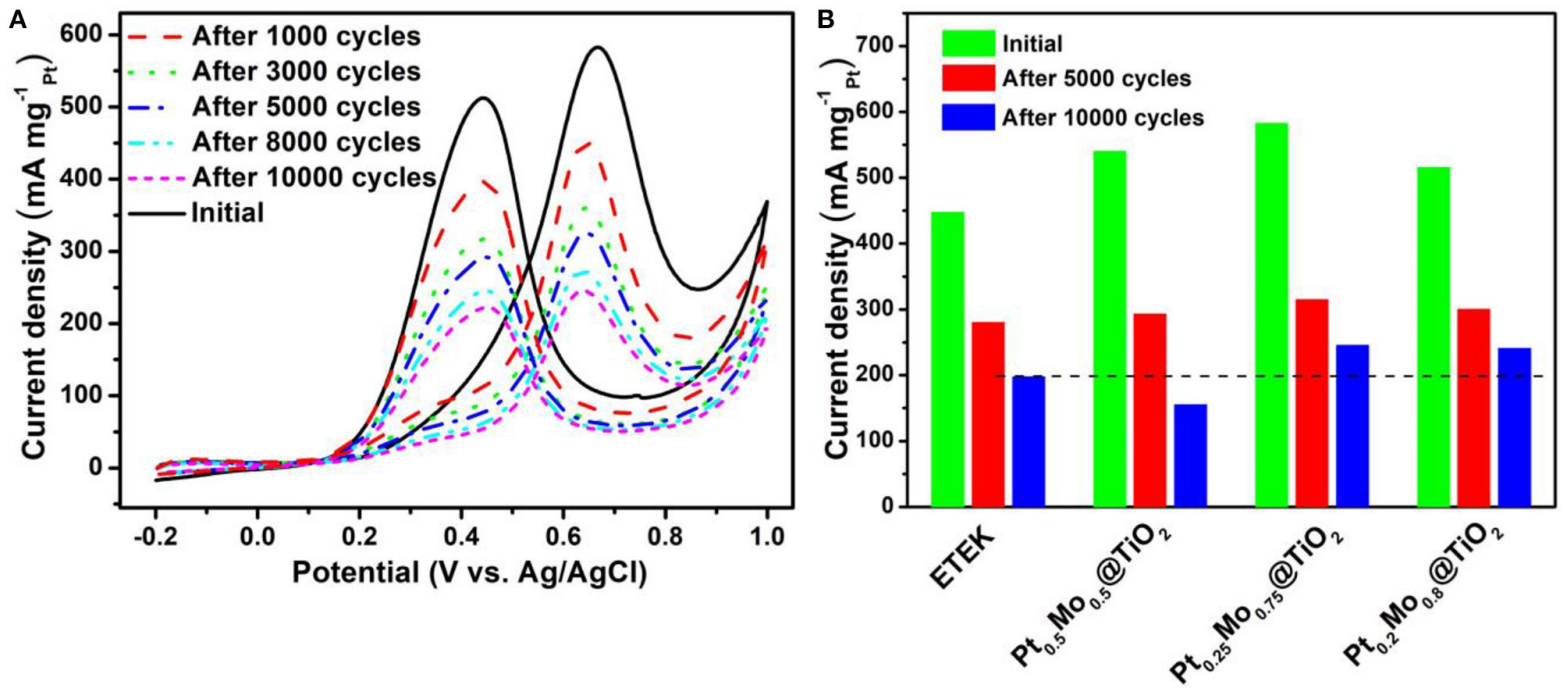
**(A)** CV curves of Pt0.25Mo0.75@TiO2 at the scan rate of 50 mV·s^−1^ in 0.5 M H_2_SO_4_ + 1.0 M CH_3_OH aqueous solution at room temperature, **(B)** Histogram of mass activities at the scan rate of 50 mV·s^−1^ in 0.5 M H_2_SO_4_ + 1.0 M CH_3_OH aqueous solution at room temperature.

The durability tests are carried out by sweeping the potential from −0.2 V to 1.0 V (vs. Ag/AgCl) for 10,000 cycles at a sweep rate of 50 mV s^−1^ as shown in Figure 7. It can be seen clearly that the current density decreases and the onset potential shifts positively with increasing the scan cycles in Figure 7A. As can be seen from Figure 7B, the mass activities of ETEK, Pt0.5Mo0.5@TiO2, Pt0.25Mo0.75@TiO2, and Pt_0.2_Mo_0.8_@TiO_2_ decrease respectively to 55.6, 72.5, 57.3, and 53.8% after 10,000 cycles. Compared to the commercial Pt/C (ETEK), the Pt0.25Mo0.75@TiO2 electrocatalyst still demonstrates significantly higher electrocatalytic activity after the longtime test. The highest stability and catalyst activity of Pt_x_Mo_y_@TiO_2_ electrocatalysts can be attributed to the unique core-shell structure and the uniform coating of PtMo alloy on the surface of acid-resistant TiO_2_. On the other hand, TiO_2_ has stronger corrosion resistance in acidic medium than carbon support, which can ehance the durability of catalyst. And TiO_2_ as support may modify the surface electronic structure of Pt, resulting in the enhanced electrocatalytic activity.

## Conclusion

In summary, PtxMoy@TiO2 nanoparticles with core-shell structure are prepared successfully using the RME method. The PtxMoy@TiO2 nanoparticles composed of acid-resistant TiO_2_ core with thin layer PtMo alloy shell. The component of the PtxMoy@TiO2 nanoparticles can be tuned by adjusting reactant concentrations. The PtxMoy@TiO2 electrocatalyst exhibits higher catalytic activity and stability for MOR performance relative to the commercial ETEK. Especially, the Pt0.25Mo0.75@TiO2 electrocatalyst has an excellent MOR performance. The high MOR activity is attributed to the unique core-shell structure, which can enhance the utilization rate of Pt and provide more active sites. In addition, the alloying effect of Pt and Mo can cause the shift of the d-band center of Pt atoms and increase the MOR activity. A feasible strategy for preparing core-shell structured nanoparticles is developed in this work. The research results may be beneficial to the further development of the electrocatalyst industry.

## Data Availability Statement

The original contributions presented in the study are included in the article/Supplementary Material, further inquiries can be directed to the corresponding author.

## Author Contributions

TA: conceptualization, methodology, data curation, formal analysis, resources, visualization, and writing-original draft preparation. SB: validation. JL: investigation, supervision, writing-reviewing and editing, project administration, and funding acquisition. All authors contributed to the article and approved the submitted version.

## Conflict of Interest

The authors declare that the research was conducted in the absence of any commercial or financial relationships that could be construed as a potential conflict of interest.
